# Reporting Quality of Meta-Analyses of Randomized Controlled Trials on Knee and Ankle Injury Prevention Programs in Football Players Using PRISMA 2020

**DOI:** 10.3390/sports13090283

**Published:** 2025-08-22

**Authors:** Spyridon Plakias, Anna Tsiakiri, Konstantinos Vassis, Chrysoula Doxani, Georgios Bakalos, Theodoros Mprotsis

**Affiliations:** 1 Department of Physical Education and Sport Science, University of Thessaly, 42100 Trikala, Greece; 2 Neurology Department, School of Medicine, Democritus University of Thrace, 68100 Alexandroupolis, Greece; atsiakir@med.duth.gr; 3 Human Performance and Rehabilitation Laboratory, Faculty of Physiotherapy, School of Health Sciences, University of Thessaly, 35132 Lamia, Greece; konstantinosvass@gmail.com; 4 Department of Biomathematics, School of Medicine, University of Thessaly, 41222 Larissa, Greece; doxani@uth.gr (C.D.); giorgos.bakalos@gmail.com (G.B.); tmprotsis@uth.gr (T.M.)

**Keywords:** soccer, PRISMA 2020, reporting adherence, systematic review, sports medicine

## Abstract

Background: Systematic reviews with meta-analyses play a critical role in synthesizing evidence on injury prevention programs in football. However, their utility depends on transparent and complete reporting, as promoted by the PRISMA 2020 guidelines. Aim: To assess the reporting quality of meta-analyses of randomized controlled trials (RCTs) on knee and ankle injury prevention programs in football players, using the PRISMA 2020 checklist. Methods: A methodological review was conducted following a preregistered protocol. Systematic searches in four databases (Scopus, Web of Science, PubMed, Cochrane) identified eligible meta-analyses including only RCTs on exercise-based prevention of knee or ankle injuries in football players. PRISMA 2020 adherence was evaluated across 52 items using a 3-point scale. Data were independently extracted and assessed by two reviewers. Results: Five meta-analyses met the inclusion criteria. Overall adherence to PRISMA 2020 was moderate (mean score = 70.38%), with substantial variability across sections. The Title (100%), Introduction (95.0%), and Discussion (90.0%) were best reported, while the Abstract (57.3%) and Other Information (47.3%) sections showed the lowest adherence. The Methods sections (74.7%) and the Results sections (74.5%) demonstrated a moderate level of adherence. Key underreported items included protocol registration, funding, data availability, and certainty of evidence. Conclusions: Despite moderate adherence, significant reporting gaps remain in meta-analyses on football injury prevention. Stricter enforcement of PRISMA guidelines is essential to improve transparency, reproducibility, and the practical impact of evidence syntheses in sports medicine.

## 1. Introduction

### 1.1. Background

Research on sports injury prevention strategies is rapidly growing, constituting a critical component of sports science [1,2]. This trend has also significantly impacted football, where medical subjects have emerged as a dominant topic among review articles on football performance analysis [3]. Football players are frequently exposed to intense physiological and biomechanical demands, rendering them highly susceptible to musculoskeletal injuries [4,5,6]. Among these, the knee and ankle joints are consistently reported as the most common anatomical sites of injury [7,8,9]. These injuries often result in time loss from training and competition, substantial healthcare costs, and long-term consequences for athletic careers and players’ psychological well-being [10,11].

Given the burden of such injuries, numerous randomized controlled trials (RCTs) have been conducted to evaluate the efficacy of exercise-based prevention programs targeting these vulnerable joints [12,13]. To synthesize evidence from such trials and provide recommendations for best practice, systematic reviews and meta-analyses serve as the highest level of evidence in the evidence-based hierarchy [14,15,16]. However, the utility of these syntheses depends heavily on the clarity, completeness, and transparency of their reporting [17,18].

To address longstanding concerns over inconsistent or incomplete reporting, the Preferred Reporting Items for Systematic Reviews and Meta-Analyses (PRISMA) Statement was developed, with the most recent update, PRISMA 2020, offering detailed guidance on how to report methods and findings in a way that facilitates reproducibility, appraisal, and uptake [19,20]. Adherence to its items has significantly improved the methodological and reporting quality of systematic reviews and for this reason is frequently recommended in journals’ “Instructions to Authors” [21,22].

### 1.2. Literature Review and Research Gap

In several fields of medicine, researchers have sought to evaluate the reporting quality of systematic reviews using the PRISMA guidelines. For example, Li et al. [23] assessed the reporting quality of COVID-19 systematic reviews and found that it needed substantial improvement, with one item being adequately reported in just 11.9% of studies, seven items ranging from 25.0% to 35.0%, and only six out of the 27 items being adequately reported in more than 70.0% of the reviews. Similarly, Nawijn et al. [24] found that systematic reviews and meta-analyses in emergency medicine adequately reported a mean of 18 out of 27 PRISMA items, with item-specific adherence rates ranging from 19.0% to 100.0% (mean = 70.0%). A slightly higher level of adherence per item (72.5%) was observed in systematic reviews in craniofacial surgery; however, this may partly reflect the fact that Pidgeon et al. [25] only evaluated items deemed applicable to each review.

Other authors have specifically investigated the reporting quality of abstracts of systematic reviews, finding even lower adherence rates. For instance, Maticic et al. [26], who analyzed abstracts of reviews in anesthesiology, reported a median adherence rate of 42.0% (i.e., 5 out of 12 items). The majority of included abstracts had total adherence scores below 50%, and none exceeded 75.0%. Similarly, in systematic review abstracts in operative dentistry, the mean adherence per item was 58.5%, with funding reported in 0.0% and registration in only 10.0% of cases [27].

In the field of sports, we identified only one similar study. Specifically, Cho and Shin [28] assessed the reporting quality of systematic reviews and meta-analyses in sports physical therapy, revealing multiple problematic areas including abstracts, registration, risk of bias in individual studies and across studies, effect size and variance calculations, additional analyses, and funding. In addition, Plakias [3], in a bibliometric analysis, briefly evaluated meta-analyses within the domain of football performance analysis, though this assessment focused on statistical methodology rather than reporting quality. That study highlighted several methodological shortcomings beyond reporting, including the inclusion of low-quality primary studies (“garbage in, garbage out”), combining data from studies with non-comparable protocols (“apples and oranges” problem), inadequate assessment and handling of heterogeneity when selecting between fixed- and random-effects models, and insufficient control for publication bias. Such issues underline that limitations in meta-analyses may extend beyond reporting transparency, reinforcing the broader relevance of improving both methodological and reporting practices in future evidence syntheses.

Collectively, these studies reveal a consistent pattern of incomplete adherence to PRISMA guidelines across diverse medical and sports-related fields, with particular weaknesses in reporting abstracts, registration details, funding sources, and certain methodological items. While overall adherence rates tend to cluster around a moderate level (approximately 65.0–75.0%), substantial variability exists between domains, suggesting that journal policies, author awareness, and field-specific norms may influence reporting quality. Notably, few studies have focused on reporting the quality of sport-specific meta-analyses, and even fewer on a single sport such as football, leaving an important gap in understanding whether these deficiencies persist in highly targeted research areas. In particular, a clear gap exists in the literature regarding PRISMA-based evaluations of reporting quality in meta-analyses focused on injury prevention programs in football players, as no such study currently exists. Given (a) the rapid growth of RCT-based research on sports injury prevention strategies, (b) the pivotal role of high-quality meta-analyses in evidence-based medicine, and (c) the high incidence and serious consequences of knee and ankle injuries among football players, it is essential to assess the adherence of meta-analyses on knee and ankle injury prevention programs to the PRISMA guidelines.

### 1.3. Aim of the Study

In this context, the present study aimed to assess the reporting quality of meta-analyses of RCTs investigating knee and ankle injury prevention programs in football players, using the PRISMA 2020 main and abstract checklist. Specifically, this study addressed the following research questions: (a) To what extent do published meta-analyses adhere to the PRISMA 2020 reporting guidelines? and (b) Which items of the checklist are well reported and which are underreported? By answering these questions, this study could contribute to identifying methodological gaps in current meta-analyses in the field of football injury prevention, highlight areas for improvement in reporting transparency, and provide guidance for researchers, reviewers, and journal editors aiming to enhance the quality and reproducibility of future systematic reviews with meta-analyses.

## 2. Material and Methods

### 2.1. Study Design and Protocol Registration

This study followed a descriptive methodological review design, focusing on evaluating the reporting quality of published meta-analyses. The unit of analysis was each eligible meta-analysis. The protocol was preregistered (date: 12 June 2025) on the Open Science Framework (OSF) and is publicly accessible at https://doi.org/10.17605/OSF.IO/N76BD (associated project: osf.io/q7rwz, accessed on 12 June 2025) in accordance with best practices for transparency and reproducibility in methodological research [29,30].

### 2.2. Eligibility Criteria

We included peer-reviewed meta-analyses that met the following criteria:

- Population: Football players (male or female, any age group).
- Intervention: Any exercise-based injury prevention program targeting the knee or ankle joint.
- Comparator: No intervention (standard training).
- Outcome: Incidence or risk of knee or ankle injury.
- Study design: Meta-analyses that included RCTs only.
- Reporting: Full-text articles published in English.

We excluded:

- Reviews without meta-analytical synthesis.
- Reviews focusing on non-football athletes or mixed sports without separate data for football.
- Reviews including non-randomized studies or observational designs.

### 2.3. Search and Selection Process

The search was conducted on June 1st and covered four databases: Scopus, Web of Science, PubMed, and the Cochrane Library. Based on the predefined eligibility criteria, the search strategy used combinations of the following terms: soccer OR football, knee OR ankle, injury OR injuries, randomized controlled trial OR RCT, and meta-analysis. The exact search string used for each database is presented in Table 1.

Articles identified through the initial search after removal of duplicates were screened by title and abstract for relevance. Full-text articles were then assessed to determine eligibility according to the criteria described above. After the initial search and removal of duplicates, two reviewers independently screened titles and abstracts for relevance. Full-text articles were then assessed to determine eligibility according to the criteria described above. Disagreements were resolved through discussion or by consulting a third reviewer when necessary.

### 2.4. Data Extraction

A structured data extraction form was developed to capture bibliographic details (authors, year, journal), targeted joint (knee, ankle, or both), number of included RCTs, and use of PRISMA statement. Each included study was also assessed using PRISMA 2020 across the domains of Title, Abstract, Introduction, Methods, Results, Discussion, and Other Information [19,20].

Although the PRISMA 2020 statement was published after two of the included meta-analyses (published in 2015 and 2016), all reviews were assessed using PRISMA 2020 to ensure consistency and comparability across studies. While we acknowledge that earlier reviews could not have adhered to PRISMA 2020 at the time of their publication, many of the core items in the updated checklist are conceptually consistent with PRISMA 2009 [18,31]. We therefore considered it appropriate to apply the most recent version of the guideline. Applying the same PRISMA version across all included reviews aligns with approaches adopted in other methodological assessments to ensure direct comparability between studies. For example, Li et al. [27] evaluated the abstracts of systematic reviews conducted during the pre-PRISMA period using the PRISMA framework.

The assessment was conducted using both the 27-item PRISMA 2020 checklist and the 12 items from the PRISMA 2020 abstract checklist. The first item of the abstract checklist was excluded, as it duplicates the first item of the 27-item checklist. Additionally, it is important to note that some items consisted of sub-items (e.g., 10a, 10b), each of which was evaluated independently. As a result, the final assessment comprised 52 distinct items (Supplementary File S1).

Each of the 52 items was independently scored by two reviewers using a three-point scale, adapted from previous methodological reviews [25,32]. This approach was preferred over the binary scale (adequate vs. not) used by other authors [27,33], as it provides more detailed information. Specifically, the scale included the following levels:

- 0 = not reported,
- 1 = partially reported/incomplete,
- 2 = fully reported/adequately detailed.

Item-level scores were summed to compute a total reporting quality score for each meta-analysis (maximum = 2 × 52 = 104 points). Reporting adherence was also analyzed separately for each PRISMA item (maximum per item = 2 × 5 = 10). Finally, the mean item scores for each PRISMA section (Title, Abstract, Introduction, Methods, Results, Discussion, Other Information) were also calculated for each meta-analysis.

To evaluate the consistency of PRISMA scoring between the two reviewers, interrater agreement was quantified using Cohen’s kappa (κ) for ordinal agreement across PRISMA items [34]. Discrepancies in scoring were resolved through consensus discussions with the participation of a third reviewer. In addition to the main reporting quality assessment, an exploratory visualization was conducted to examine potential relationships between total PRISMA adherence scores and selected characteristics of the included meta-analyses. In particular, a scatterplot was generated in Power BI (Office 365, Microsoft Corporation, Redmond, WA, USA, Power BI Desktop 2025). Power BI is a data visualization and analytics tool commonly used for the graphical representation and exploration of quantitative data [35,36].

### 2.5. Ethics

Ethical approval was not required for this study, as it involved the analysis of data extracted exclusively from previously published meta-analyses. No individual patient data were collected or analyzed. The study did not involve human participants, interventions, or identifiable personal information.

## 3. Results

### 3.1. Study Selection

**Figure 1:**
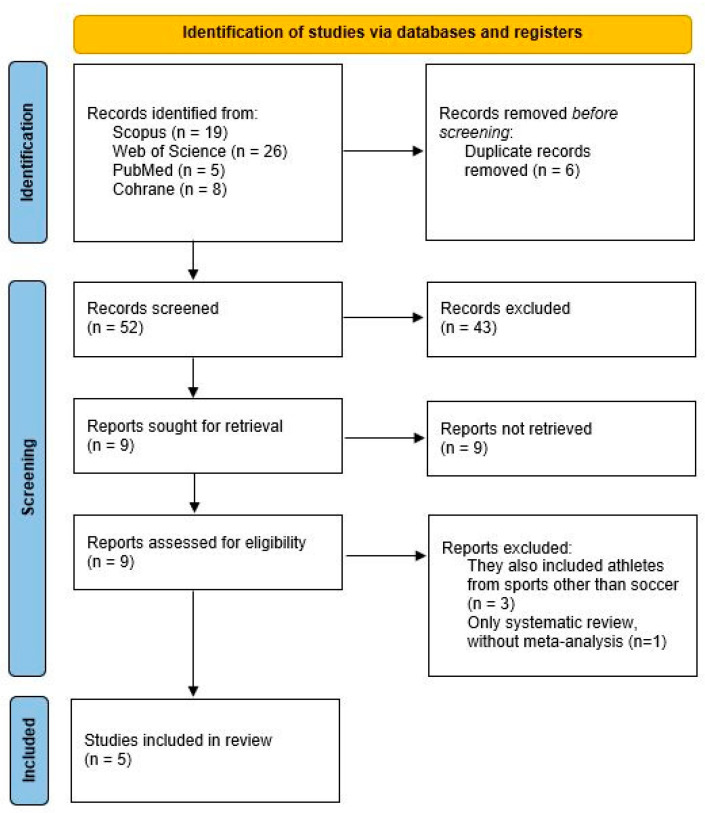
Flow diagram illustrating the study selection process.

A total of 58 meta-analyses were retrieved from the four databases, of which six were duplicates. The remaining 52 studies were screened by title and abstract based on the predefined inclusion and exclusion criteria. This screening process was conducted independently by two reviewers, and any discrepancies were resolved through discussion and consensus, involving a third reviewer when necessary. Based on this screening, nine articles were deemed eligible for full-text assessment. Ultimately, three articles were excluded because they included athletes from other sports in addition to football [37,38,39] and one because it was only a systematic review without a meta-analysis [40]. As a result, five systematic reviews with meta-analyses were included in the present methodological review [7,41,42,43,44]. The entire article selection process is illustrated in the flow diagram (Figure 1).

### 3.2. Characteristics of Included Meta-Analyses

Table 2 presents the characteristics of the meta-analyses included in our study, arranged in chronological order of publication. As shown in the table, three out of the five reviews focused on the knee (two on the ankle). Three of the five reviews followed the PRISMA 2020 guidelines, while the remaining two adhered to PRISMA 2009. The number of RCTs included in each meta-analysis ranged from nine to eleven.

### 3.3. PRISMA Adherence

**Figure 2:**
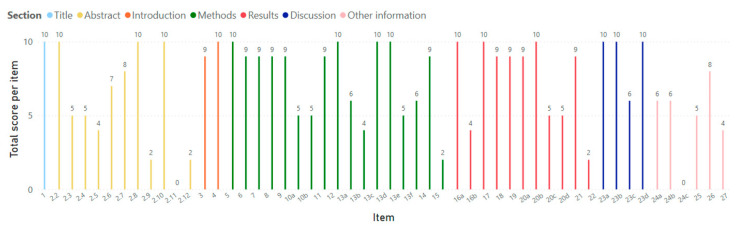
Distribution of total adherence scores (0–10) for each individual PRISMA 2020 item, with color-coded grouping by checklist section.

Supplementary File S2 presents the scoring outcomes of the reporting quality assessment for the five included meta-analyses based on 52 distinct PRISMA 2020 items. Each item corresponds to a specific element of the PRISMA checklist and is grouped by manuscript section (e.g., Title, Abstract, Introduction, Methods, Results, Discussion, Other Information). For each item, individual scores from the five meta-analyses are reported, along with the total score per item and the average score per section. At the bottom, total reporting scores for each paper are also shown. Figure 2 visually presents the total score for each PRISMA item. Items belonging to the same section are shown in the same color, while different sections are represented by different colors.

Overall, reporting adherence varied substantially across both individual items and sections. The highest mean section score was observed in the Title section (mean = 10.0), followed closely by the Introduction (mean = 9.5) and Discussion (mean = 9.0) sections. In contrast, the Other Information section demonstrated the lowest adherence (mean = 4.8), with several items such as funding, competing interests, and data availability often being omitted or only partially addressed.

The Methods and Results sections yielded intermediate mean scores (7.5 and 7.4, respectively), although several sub-items within these sections (e.g., risk of bias due to missing results, certainty of evidence) were inconsistently reported. The Abstract section showed moderate adherence (mean = 5.7), with variation in the completeness of reporting across individual abstract components.

Total reporting quality scores per paper ranged from 63 to 83 out of a maximum 104 (mean = 73.2, standard deviation = 7.3). Therefore, the average reporting adherence of the five articles was (73.2/104)×100% = 70.4%. It is worth noting that for the three studies conducted after 2020 (and following PRISMA 2020) the average adherence was 72.4%, while for the two studies conducted before 2020 the average adherence was 67.3%. Finally, the items that were not reported at all in any of the five articles were 2.11 and 24c, while items 2.12, 15, and 22 were reported in only one article.

**Figure 3:**
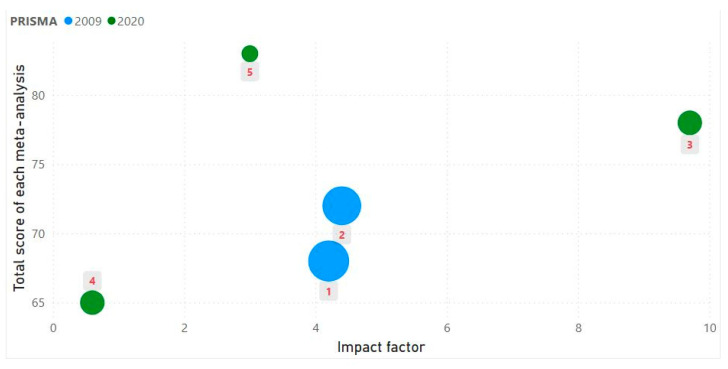
Relationship between journal impact factor and total PRISMA adherence score for each included meta-analysis. The numbers next to the bubbles indicate which study each bubble refers to, and the numbering of the studies follows Table 2.

The scatterplot in Figure 3 illustrates the relationship between the impact factor of the publishing journal (x-axis) and the total PRISMA adherence score of each meta-analysis (y-axis). The color of each point indicates whether the meta-analysis followed PRISMA 2009 (blue) or PRISMA 2020 (green). The size of each bubble reflects the recency of the study, calculated as 2025 minus the year of publication. The red number inside each gray label represents the study ID as referenced in Table 2. A visual inspection of Figure 3 suggests that meta-analyses adhering to the PRISMA 2020 guidelines generally achieved higher total reporting scores than those using PRISMA 2009. Furthermore, there appears to be a modest positive trend between journal impact factor and reporting quality. However, no consistent pattern was observed in relation to publication recency. While the limited sample size (n = 5) precludes formal inferential testing, the visual patterns in Figure 3 may indicate that both PRISMA version and journal impact factor could partly explain variability in reporting quality. However, given the extremely small dataset, any such relationships should be interpreted with great caution and considered only as preliminary observations rather than evidence of statistical association.

## 4. Discussion

### 4.1. Overview

Knee and ankle injuries remain among the most prevalent injuries in football, leading to substantial time loss, healthcare costs, and potential long-term consequences for players. Although numerous RCTs have investigated the effectiveness of injury prevention programs for these joints, the extent to which such evidence is transparently and comprehensively reported in meta-analyses remains unclear. This gap in the literature underscores the importance of evaluating the reporting quality of meta-analyses in this area, as high-quality reporting is essential for reproducibility, clinical translation, and policy-making. In this context, the present methodological review aimed to assess the adherence of meta-analyses on knee and ankle injury prevention programs in football players to the PRISMA 2020 guidelines. The overall adherence was moderate (70.4%), with notable variation across checklist items and sections. While the Title, Introduction, and Discussion were generally well reported, significant deficiencies were observed in the Abstract and Other Information sections. The Methods and Results sections demonstrated intermediate levels of adherence. Several critical items, such as funding disclosures, data availability, and assessments of evidence certainty, were consistently underreported or omitted entirely. These findings highlight ongoing challenges in transparent and complete reporting, even in high-level evidence syntheses within sports medicine.

### 4.2. Adherence of Meta-Analyses to the PRISMA 2020 Guidelines

The overall adherence of the included meta-analyses to the PRISMA 2020 reporting guidelines was moderate (mean = 70.4%). This finding aligns with previous studies in related fields, such as craniofacial surgery [25], emergency medicine [24], and sports physical therapy [28], where adherence ranged from approximately 70.0% to 75.0%. By contrast, COVID-19-related systematic reviews evaluated by Li et al. [23] demonstrated substantially lower adherence (mean of 14 out of 27 PRISMA items), likely due to the urgency and volume of publications during the pandemic, which prioritized rapid dissemination over strict compliance with reporting guidelines.

Notably, even meta-analyses published after the release of PRISMA 2020 did not consistently comply with the updated items. In our review, adherence scores ranged from 68.0% to 83.0%, suggesting that journal policies and editorial practices may play a key role in how strictly PRISMA compliance is enforced. This observation is supported by Fleming et al. [32], who found that PRISMA adherence was significantly associated with both the journal of publication and the number of contributing authors. Moreover, reviews published in the Cochrane Database of Systematic Reviews achieved significantly higher PRISMA scores than those published in conventional journals.

Finally, it is important to recognize that reporting quality, as defined by PRISMA, is only one aspect of a high-quality systematic review with meta-analysis. Fleming et al. [45] warned that excessive reliance on PRISMA could lead reviewers to overlook other methodological standards, such as MOOSE for observational studies, QUADAS-2 for diagnostic accuracy studies, or AMSTAR for evaluating methodological quality. They also emphasized the value of involving methodologists and including a meta-analysis, both of which were associated with more appropriate guideline use.

In parallel, Basile et al. [46] argued that the credibility of a meta-analysis depends not only on reporting practices but also on fundamental methodological considerations, such as the quality of the primary studies, the biological plausibility of the research question, the level of heterogeneity, and the application of robust statistical methods, including meta-regression, trial sequential analysis, and individual participant data (IPD) synthesis. They also highlighted that methodological flaws, such as the inclusion of low-quality studies or failure to address publication bias, can significantly compromise the validity of the results.

### 4.3. Well-Reported and Underreported PRISMA Sections and Items

The detailed assessment of individual PRISMA items revealed substantial variability in reporting completeness across the five included meta-analyses. Section-wise, the Abstract and Other Information domains demonstrated the lowest average adherence scores. Similarly low compliance for Abstract items (approximately 42.0%) was also reported by Peters et al. [33] and Maticic et al. [26], who evaluated the reporting quality of systematic review abstracts using the 12-item PRISMA-A checklist. One potential explanation may be the strict word limits imposed by many journals on abstracts, which constrain authors from fully addressing all required elements. This raises the question of whether journal editors should reconsider such restrictive practices, especially if they concurrently expect authors to comply with PRISMA standards. Alternatively, it may be worth revisiting the PRISMA-A checklist itself for specific items. For instance, Item 3 (“Specify the inclusion and exclusion criteria for the review”) could be refined to include the word “main,” as expecting a complete list of criteria in an abstract may be excessive, particularly when this information typically occupies an entire table in the main text of the article.

As for the remaining sections, Other Information was newly introduced in the PRISMA 2020 update and was not present in the 2009 version. Li et al. [47], who evaluated systematic reviews using the updated PRISMA 2020, also found low compliance for this section (mean adherence = 50.8%), supporting the results of the present review. In contrast, adherence was generally high in the Introduction and Discussion sections and moderate in the Methods and Results sections, patterns consistent with previous PRISMA evaluations [24,25,28,32,33,47].

A more granular item-level analysis revealed that within the Abstract, the least reported elements were those pertaining to limitations, funding, registration, eligibility criteria, information sources, and risk of bias. Similar deficiencies were noted by both Maticic et al. [26] and Peters et al. [33] in their respective assessments. However, Peters et al. [33] observed that Cochrane reviews exhibited better abstract reporting overall, with deficiencies mainly limited to the reporting of risk of bias, funding, and registration.

Regarding the main text of the meta-analyses, the most underreported items (scoring below 5 on the 0–10 scale) were those related to: (a) methods used to prepare or process data; (b) assessment of the certainty of evidence; (c) listing and explaining excluded studies that initially appeared eligible; (d) describing any protocol deviations or amendments; and (e) availability of data, code, and materials. The consistently low reporting of these key items may reflect a combination of factors, including limited journal space, insufficient author awareness of these reporting requirements, and variability in editorial enforcement of PRISMA standards. Interestingly, the items related to risk of bias were well reported across all five meta-analyses in this review. This contrasts with prior findings, where significant gaps in reporting these items were identified [23,24,25,28,32,47]. A plausible explanation may be that those prior studies included systematic reviews both with and without meta-analysis, whereas the current review focused exclusively on meta-analyses.

Taken together, these findings suggest that while authors generally strive to meet PRISMA requirements, and often succeed to a satisfactory extent, critical elements tied to transparency, reproducibility, and methodological appraisal (e.g., funding sources, protocol registration, data availability, and certainty of evidence) are frequently overlooked. Improving these aspects is essential to enhance the trustworthiness, interpretability, and overall scientific value of systematic reviews and meta-analyses in the field of sports medicine.

### 4.4. Strengths and Limitations of the Present Review

This study has several strengths. First, to our knowledge, it is the first methodological review specifically evaluating the reporting quality of meta-analyses on knee and ankle injury prevention programs in football players using the updated PRISMA 2020 checklist. Second, a comprehensive and reproducible search strategy was applied across four major databases, and all included studies were systematically assessed using a detailed 52-item framework derived from the main and abstract PRISMA 2020 checklists. Third, the evaluation process involved independent scoring by two reviewers, with strong inter-rater agreement (Cohen’s κ = 0.8) and resolution of discrepancies through consensus with a third reviewer, enhancing the reliability of the results.

Nonetheless, this review has certain limitations. The sample size was limited to five eligible meta-analyses, reflecting the relative scarcity of such studies in the specific domain of football injury prevention. Unlike previous studies that employed inferential statistics to explore associations between reporting quality and bibliometric characteristics [48], the present review did not apply formal statistical testing due to the very limited sample size (n = 5). Instead, an exploratory scatterplot was generated to visually examine potential trends between the PRISMA total score of each meta-analysis and selected variables, including year of publication, PRSMA version, and the impact factor of the publishing journal. While these visualizations offer preliminary insights, they should be interpreted with caution and not considered evidence of statistical associations. Additionally, two of the included reviews were published before the release of PRISMA 2020; although the use of the updated checklist ensured consistency, it may have introduced a conservative bias in their scoring. Finally, the study assessed only the reporting quality and not the underlying methodological rigor or risk of bias of the included meta-analyses, which may also impact their utility in evidence-based decision-making.

### 4.5. Practical Implications and Directions for Future Research

The findings of this review underscore the importance of improving adherence to PRISMA reporting standards in meta-analyses addressing injury prevention in football. Transparent and comprehensive reporting facilitates critical appraisal, replication, and uptake of evidence into clinical practice and policy. Key elements, such as protocol registration, funding statements, data availability, and assessments of evidence certainty, should be routinely included, as their absence compromises both the credibility and usability of the research. To facilitate consistent adherence to PRISMA 2020, journals and publishers could integrate practical mechanisms into the publication workflow. These may include the mandatory submission of completed PRISMA checklists at the time of manuscript submission, automated screening tools to flag incomplete reporting, and structured peer review templates that specifically address PRISMA items. Such measures, in combination with author training initiatives, could help embed reporting standards into routine scholarly practice, ultimately improving the transparency and reproducibility of meta-analyses in sports medicine.

Importantly, poor adherence to PRISMA reporting standards does not merely reflect a methodological shortcoming, but also can have clinical consequences [24,49]. When systematic reviews and meta-analyses lack transparency in areas such as protocol registration, funding disclosures, or certainty of evidence, clinicians and policy-makers may be misled by incomplete or selectively reported findings [22,50]. This can influence decision-making regarding injury prevention programs, potentially leading to the implementation of ineffective or suboptimal strategies. For example, the absence of protocol registration may allow selective outcome reporting, while failure to assess the certainty of evidence could result in overreliance on low-quality findings, ultimately compromising injury prevention efforts in practice. In fields like sports medicine, where evidence is directly translated into athlete care and training protocols, such gaps may result in missed opportunities for injury reduction or even unintended harms [51]. Therefore, enhancing reporting quality is not just a matter of academic rigor but a prerequisite for safe and effective clinical translation.

Future research should aim to: (a) increase awareness and enforcement of PRISMA 2020 guidelines among authors, reviewers, and journal editors in the field of sports medicine; (b) examine the methodological quality and risk of bias of existing meta-analyses in addition to their reporting quality; and (c) expand the scope of reporting quality assessments to other injury sites, sports, or populations. Journals can contribute to these efforts by explicitly requiring PRISMA compliance in their submission guidelines and providing authors with tools or checklists to assist during manuscript preparation. Finally, replication of a similar review with a larger sample could confirm current patterns and inform targeted interventions to improve reporting practices.

## 5. Conclusions

This methodological review assessed the reporting quality of meta-analyses on knee and ankle injury prevention programs in football players using the PRISMA 2020 statement. Overall, the findings indicate moderate adherence to PRISMA guidelines, with substantial variation across checklist items and manuscript sections. While certain components, such as the Title, Introduction, and Discussion, were generally well reported, critical elements related to transparency, such as protocol registration, funding disclosures, data availability, and certainty of evidence, were frequently underreported or omitted altogether.

These results underscore the ongoing need for improved reporting practices in meta-analyses within sports medicine. Researchers are encouraged to systematically apply the PRISMA 2020 checklist throughout all stages of manuscript preparation, while journal editors and reviewers should enforce adherence as part of the peer review process. Enhancing reporting quality is not only essential for ensuring the reproducibility and trustworthiness of evidence syntheses but also for facilitating their translation into effective injury prevention strategies in football.

Future efforts should expand the scope of PRISMA-based evaluations to include other types of injuries and sports, as well as assess methodological quality alongside reporting quality. Ultimately, strengthening both the conduct and reporting of meta-analyses can contribute to more informed, evidence-based decision-making in sports health and performance contexts.

## Figures and Tables

**Table 1:** Detailed search strategies applied across the four databases.

Database	Search String
Scopus	(TITLE-ABS-KEY (meta-analysis OR “meta analysis” OR metaanalysis) AND TITLE-ABS-KEY (“randomized controlled trial” OR rct) AND TITLE-ABS-KEY (injury OR injuries) AND TITLE-ABS-KEY (ankle OR knee) AND TITLE-ABS-KEY (soccer OR football))
Web of Science	meta-analysis OR “meta analysis” OR metaanalysis (All Fields) and “randomized controlled trial” OR rct (All Fields) and injury OR injuries (All Fields) and ankle OR knee (All Fields) and soccer OR football (All Fields)
PubMed	((((meta-analysis OR meta analysis OR metaanalysis) AND (randomized controlled trial OR rct)) AND (injury OR injuries)) AND (ankle OR knee)) AND (soccer OR football)
Cohrane	“meta-analysis” OR “meta analysis” OR “metaanalysis” in Title Abstract Keyword AND “randomized controlled trial” OR “rct” in Title Abstract Keyword AND “ankle” OR “knee” in Title Abstract Keyword AND “injury” OR “injuries” in Title Abstract Keyword AND “soccer” OR “football” in Title Abstract Keyword—(Word variations have been searched)

**Table 2:** Characteristics of the meta-analyses included in the study, arranged in chronological order of publication.

ID	Authors	Year	Journal (Impact Factor)	Joint	Included RCTs	PRISMA
1	Grimm et al. [42]	2015	The American Journal of Sports Medicine (4.2)	Knee	9	2009
2	Grimm et al. [41]	2016	The Journal of Bone and Joint Surgery (4.4)	Ankle	10	2009
3	Al Attar et al. [7]	2022	Journal of Physiotherapy (9.7)	Ankle	9	2020
4	Al Attar et al. [43]	2022	Isokinetics and Exercise Science (0.6)	Knee	9	2020
5	Magaña-Ramírez et al. [44]	2024	Journal of Science and Medicine in Sport (3.0)	Knee	11	2020

## Data Availability

The data are available in Supplementary File S2.

## Supplementary Materials

The following supporting information can be downloaded at: https://www.mdpi.com/article/10.3390/sports13090283/s1, File S1: Full list of PRISMA 2020 items included in the evaluation of reporting quality; File S2: Reporting quality scores of the five included meta-analyses across 52 PRISMA 2020 items.
