# Supplementary material for: Reporting Quality of Meta-Analyses of Randomized Controlled Trials on Knee and Ankle Injury Prevention Programs in Football Players Using PRISMA 2020
Source: Sports (Basel). 2025 Aug 22;13(9):283. doi: 10.3390/sports13090283 (PMC12473353; doi:10.3390/sports13090283)
Supplement: Supplementary file 1 [file sports-13-00283-s001.zip › Supplementary file S2.pdf]

Reporting quality scores of the five included meta-analyses across 52 PRISMA 2020 items

Section	Item	ID 1	ID 2	ID 3	ID 4	ID 5	Total score per item	Mean per section
TITLE	1	2	2	2	2	2	10	10.00
ABSTRACT Background	2.2	2	2	2	2	2	10	
	2.3	1	1	1	1	1	5	
ABSTRACT Methods	2.4	1	2	0	1	1	5	
	2.5	2	2	0	0	0	4	
	2.6	1	2	2	1	1	7	5.73
ABSTRACT Results	2.7	2	2	2	1	1	8	
	2.8	2	2	2	2	2	10	
ABSTRACT Discussion	2.9	0	1	0	0	1	2	
	2.10	2	2	2	2	2	10	
ABSTRACT Other	2.11	0	0	0	0	0	0	
	2.12	0	0	2	0	0	2	
INTRODUCTION	3	2	2	1	2	2	9	9.50
	4	2	2	2	2	2	10	
METHODS	5	2	2	2	2	2	10	7.47
	6	2	2	1	2	2	9	
	7	2	2	2	1	2	9	
	8	2	2	2	1	2	9	
	9	2	2	2	1	2	9	
	10a	1	1	1	1	1	5	
	10b	1	1	2	1	1	5	
	11	2	2	2	1	2	9	
	12	2	2	2	2	2	10	
	13a	1	1	2	1	1	6	
	13b	0	1	1	1	1	4	
	13c	1	2	2	2	2	10	
	13d	2	2	2	2	2	10	
	13e	0	0	2	1	2	5	
	13f	1	2	0	1	2	6	
	14	2	2	1	2	2	9	
	15	0	0	0	0	2	2	
	16a	2	2	2	2	2	10	
RESULTS	16b	0	1	2	1	0	4	7.45
	17	2	2	2	2	2	10	
	18	2	2	2	1	2	9	
	19	2	2	2	1	2	9	
	20a	2	2	2	1	2	9	
	20b	2	2	2	2	2	10	
	20c	0	0	2	1	2	5	

	20d	1	2	0	0	2	5	
	21	2	2	1	2	2	9	
	22	0	0	0	0	2	2	
	23a	2	2	2	2	2	10	
DISCUSSION	23b	2	2	2	2	2	10	9.00
	23c	2	1	1	1	1	6	
	23d	2	2	2	2	2	10	
	24a	0	0	2	2	2	6	
	24b	0	0	2	2	2	6	
OTHER INFORMATION	24c	0	0	0	0	0	0	4.83
	25	1	0	2	1	1	5	
	26	2	0	2	2	2	8	
	27	0	0	2	0	2	4	
Total score per paper		68	72	78	65	83		
